# Ensuring Equity, Diversity, and Inclusiveness in Genetic Analysis Will Empower the Future of Precision Medicine∗

**DOI:** 10.1016/j.jacadv.2023.100769

**Published:** 2023-12-14

**Authors:** Keith R. Brunt, Victoria Northrup

**Affiliations:** a Department of Pharmacology, Dalhousie University, Halifax, Nova Scotia, Canada; b Dalhousie Medicine New Brunswick, Saint John, New Brunswick, Canada; c Departments of Cardiology & Cardiac Surgery, New Brunswick Heart Center, Saint John Regional Hospital, Saint John, New Brunswick, Canada

**Keywords:** cardiomyopathy, genetic testing, gene variant classification, race

**Figure:**
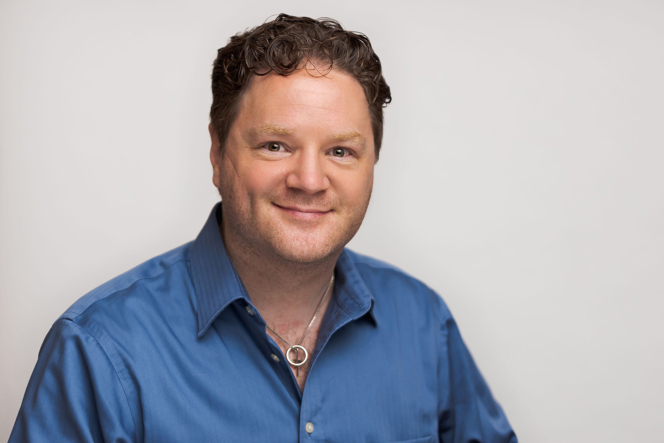

Cardiovascular disease is a spectrum of heritable and acquired exposures driving complex disease presentations. Reducing disparity is a collective effort we should all embrace to achieve clinical precision. Genetic testing is a powerful clinical tool. It can reconcile heritable traits with cardiovascular diseases, like cardiomyopathies, as part of standard medical management.^1^, ^2^, ^3^, ^4^ There is a persisting risk of variant misclassification as validated pathogenicity is informed iteratively by new information.^5^ Inaccurate variant classification can result in false alarm/reassurance. Prior studies showed 5 hypertrophic cardiomyopathy-associated variants with higher minor allele frequency (MAF) in Black Americans compared to White Americans resulting in downgraded pathogenicity.^5^ Golbus et al^6^ established a wide variation of allele frequencies in 3 cardiomyopathy-associated genes in African, American, Asian, and European ancestries.

Ancestry has a significant impact on allele frequencies and classification. Genetic studies elevate our understanding of pathogenic variants and their associated molecular pathways and present new therapeutic targets. Despite a substantial increase in genomic studies, the heavy bias toward European ancestry hinders clinical utility and reliability. This ancestry accounts for 80% to 90% of genomic studies,^5^^,^^7^, ^8^, ^9^ whereas European descent is only ∼15% of the world’s population (Figure 1A).^10^ Even in countries with diverse urban populations, genomic studies remain heavily biased toward European ancestry. Multiple factors contribute to this.

**Figure 1:**
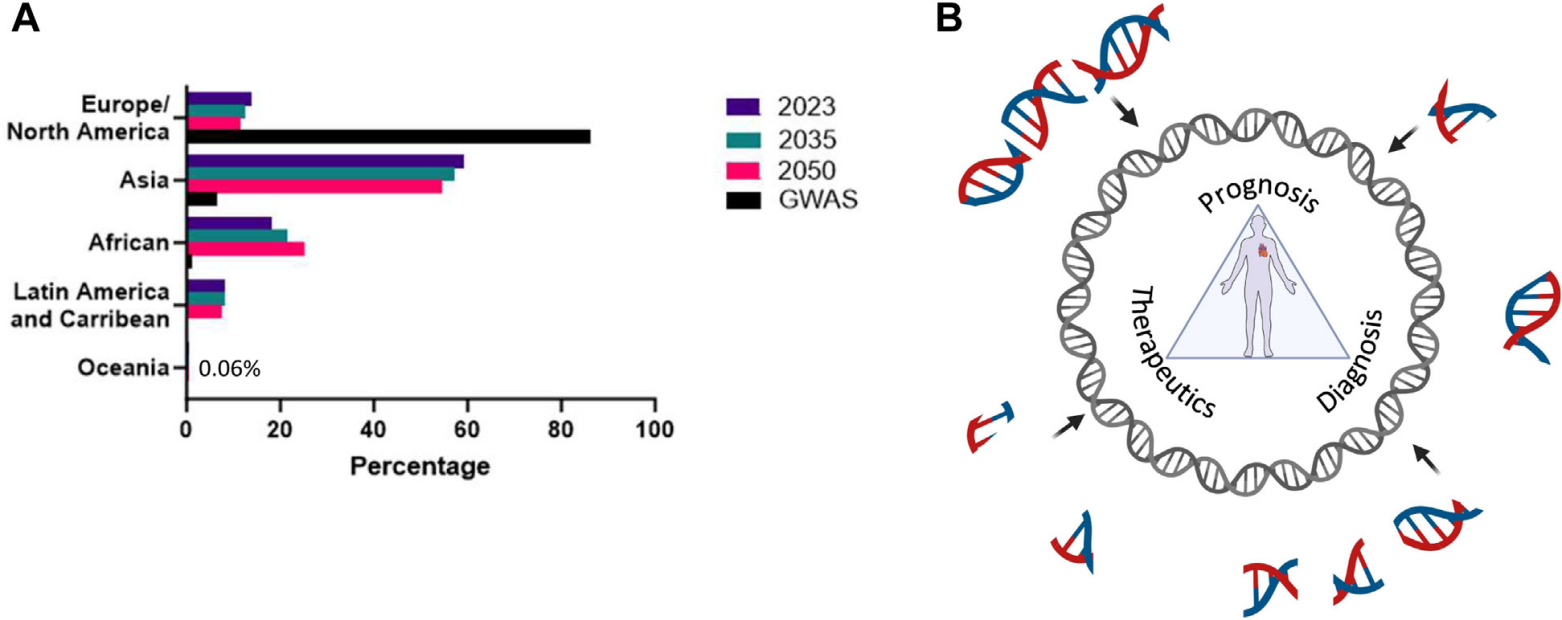
Diversity of Genomic Studies Improves Precision Medicine (A) World population projections compared to genome-wide association studies (GWAS). (B) State-of-the-art known human variation (outer circle) is fragmented into what is needed (inner circle, gray) for precision medicine.

Large-scale genomic studies are sophisticated and costly—capacity abundant in Europe and North America (colonized foremost by Europeans). Underrepresented or marginalized populations experienced historical injustices, coercion, or deception, driving negative experiences and distrust.^7^ Low visible representation and unconscious bias of who is approaching and who is approached for study enrollment. Low genomic diversity leads to inaccurate allele frequency estimates, as European allele frequencies dominate risk assignment and are unlinked to evolutionary events such as human migration, ecological assimilation, or diversification that are physiological.

As genetic tests are to be considered in an ancestry context, this can perpetuate healthcare disparities, especially when pathogenicity is conflated with race or ethnicity.^11^ Social constructs are ill-suited for pathogenetic diagnosis or differentials, and negative outcomes associated with racial context are more likely caused by racist actions, social determinants, or systemic racism perpetrating harm to patients.^12^ It is important to distinguish biologically determined pathogenetic variation, sex determinism, and epigenetics into reference data or annotations that inform the intersectionality of genetic diagnosis, counseling, and medical management.^3^^,^^13^^,^^14^ Reconciling the predominance of male/White, first and foremost in clinical trials and research data collections requires conscientious action to achieve equity.^15^

Thankfully, cardiologists and clinical geneticists are making antiracism progress medically by focusing attention on expanding ancestral diversity of genetic reference and variant risk (re)assignment, so as to be more inclusive of the diverse populations we seek to serve.^12^ Rosamilia et al^16^ examined the instability of variant classification in cardiomyopathy-associated genes including 10 sarcomeric and 6 desmosomal genes from the ClinVar database between 2011 and 2021. Cardiomyopathy-associated variants in ClinVar were reevaluated at a higher rate compared to other variants in ClinVar (2.34 times/10 y in cardiomyopathy vs 2.03 times/10 y average). Importantly, 7.3% of the cardiomyopathy-associated variants underwent a clinically significant category change (compared to 4.4% average for all variants). These results suggest an increased instability in the categorization of cardiomyopathy-associated variants. A change in categorization could result in inaccurate diagnosis and prognostic information for patients. A pathogenic or likely pathogenic variant can be stressful to an individual and their family, resulting in additional testing, radical changes to lifestyle, or medical management (under-over-treatment).

Rosamilia et al^16^ further evaluated the potential reasons for the lower stability in classification of these variants and found that variation in MAF between populations resulted in the downgrading of 29% of the variants that were reevaluated. MAF is utilized in the categorization of variants as the typically pathogenic are very low MAF and higher MAFs are typically considered benign.^17^ Many of the variants that decreased in pathogenicity had a MAF of <0.0001 in European ancestry, suggesting higher pathogenicity. Yet, the MAF in non-European ancestry was >0.0001, indicative of benign variation. Ancestry was even more significant in these downgrades compared to variant evidence quality. Given the overrepresentation of European ancestry in genetic studies, this biases MAF to those of European populations. Therefore, variants with MAF that are higher in non-European populations may not be captured and result in increased pathogenicity classifications. Taken together, potential harm to patients of both European and non-European ancestry is implicated.

Increasing diversity in genomic studies will allow for improved recognition of natural and evolutionary genetic diversity. This will result in accurate variant classification by accounting for diversity in population genetics (Figure 1B). One limitation of the Rosamali et al^16^ study is that they did not examine temporal variations. During the period of the study, the classification framework was updated in 2015.^17^ Therefore, it is possible that accuracy is already improving as awareness of studies of priority populations grows. Regardless, continuing to increase our diversity in genomic studies will lead to better and more equitable healthcare for all patients. The variant classification instability shown by Rosamilia et al^16^ also highlights the need for clinical reviews of patients that have already received genetic testing. Particularly for patients that showed sarcomeric variation, a significant number were downgraded from pathogenic or likely pathogenic to a variant of unknown significance. Patient reviews could reconcile unnecessary medical management (for the patient or any relatives). A downgrade from a variant of unknown significance to benign is less likely to change medical management and therefore will be less significant. Rosamilia et al^16^ did not examine how these changes would affect medical management for the patient or their relatives. However, this study^16^ does support the need for patient review as suggested by the American Heart Association.^1^

As we improve the accuracy of genetic testing with increased diversity of genetic studies, we should reflect on the non-European ancestry as a percentage of the world population compared to genome-wide association studies completed to date. If we do not increase diversity in genomic studies, the disparity will worsen (Figure 1). Just as the issue of bias in genetic studies results from multiple factors, the solutions to improving diversity are also multifaceted. Fatumo et al^7^ and Lemke et al^9^ advise steps toward improving diversity. Briefly, they recommend collaborations that focus funding on establishing or expanding global capacity within various underrepresented regions. Building capacity will also improve engagement with marginalized communities in North America. In addition to scientific capacity, collaborations should include expertise in ethical, legal, and social implications of genomic research. This will improve accountability and allow for appropriate data sharing and annotations. By engaging with local communities as patient partners^18^^,^^19^ and encouraging collaboration, we can secure diverse representation globally. This should include connecting meaningfully with Indigenous communities to better understand their needs and identifying diaspora in urban enclaves, or high-density settler founder-effect subpopulations in North America and Europe.

Rosamilia et al^16^ focused on rare monogenic variants. Yet, polygenic variants can be utilized to identify individuals at risk of cardiomyopathy or other hereditary risk factors for cardiovascular disease without a known dominant pathogenic variant.^4^^,^^20^ Identification of polygenic risk factors typically requires large genomic datasets with excellent phenotypic data. Polygenic risk scores were developed using data biased toward European descent, thus offering limited or inaccurate scores with low clinical utility, particularly for those of non-European or mixed ancestry. Advancing these scores with currently available reference data could further exacerbate health disparities for all.^21^ Polygenic risk is a product of quality in heterogenous reference.

In addition to the accuracy of diagnosis and prognosis, many genetic studies are used to identify pathways and develop novel therapeutic targets. With a large portion of the global population lacking representation, this research-informed utility is highly restricted, undermining effective therapeutic development. To address these challenges and ensure that all cardiomyopathy patients benefit from advances in genetic research, there's a clear need for more inclusive and representative studies. This involves collaboration, ethical practices, data sharing, and a concerted effort to engage with diverse communities. By doing so, we can work toward a future where genetic information is harnessed to its fullest potential, benefiting individuals from all walks of life and reducing health disparities.

## Funding support and author disclosures

Dr Brunt was funded by the Natural Sciences and Engineering Research Council (NSERC) [grant # RGPIN-(2020)-04878]. Dr Northrup was supported by the NSERC Alexander Graham Bell Canada Graduate Scholarship-Doctoral (CGS D).
